# Supplementary material for: Integrin-mediated electric axon guidance underlying optic nerve formation in the embryonic chick retina
Source: Commun Biol. 2023 Jun 30;6:680. doi: 10.1038/s42003-023-05056-x (PMC10313674; doi:10.1038/s42003-023-05056-x)
Supplement: Supplementary file 2 — Supplementary Information [file 42003_2023_5056_MOESM2_ESM.pdf]

Supplementary materials for

Integrin-mediated electric axon guidance underlying optic nerve formation in the embryonic chick retina

Masayuki Yamashita

E-mail: my57@iuhw.ac.jp

This PDF file includes:
Supplementary Figures 1 to 13

Supplementary Figure 1

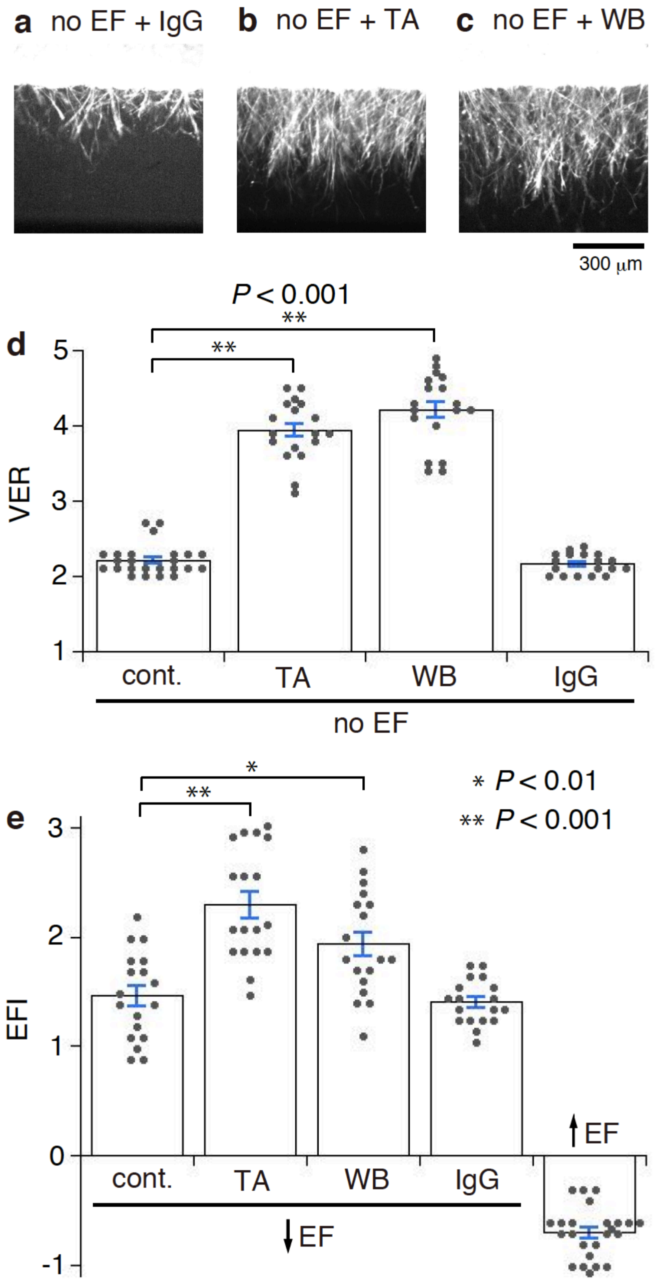

Supplementary Fig. 1: TASC and W1B10 increase the ventral extension of RGC axons both with and without EF.

a RGC axons extending from the ventral edge at the central part of retinal strip cultured without EF in the presence of the negative control isotype antibody mouse IgG1 (100 μg/mL). **b** RGC axons cultured without EF in the presence of TASC (100 μg/mL). **c** RGC axons cultured without EF in the presence of W1B10 (100 μg/mL). **d** VERs (mean ± s.e.m.) of retinal strips cultured without EF in the presence of TASC (100 μg/mL) (TA), W1B10 (100 μg/mL) (WB), and the control antibody (100 μg/mL) (IgG). Each column represents the value obtained from 18 photos taken at different focus levels from 3 retinal strips. The control without drug (cont.) was replotted from Fig. 1k (no). **e** EF index (EFI) indicates [(VERs with

EF) - (mean VER without EF)]. EFIs (mean ± s.e.m.) of retinal strips cultured in the ventrally directed EF (downward arrow) without drug (cont.), with TASC (TA), W1B10 (WB), and the control antibody (IgG), and in the dorsally directed EF without drug (upward arrow). These data were obtained from **d** and Fig. 1k. Horizontal bars and asterisks denote significant differences (two-tailed *t*-test, * $P < 0.01$, ** $P < 0.001$).

Supplementary Figure 2

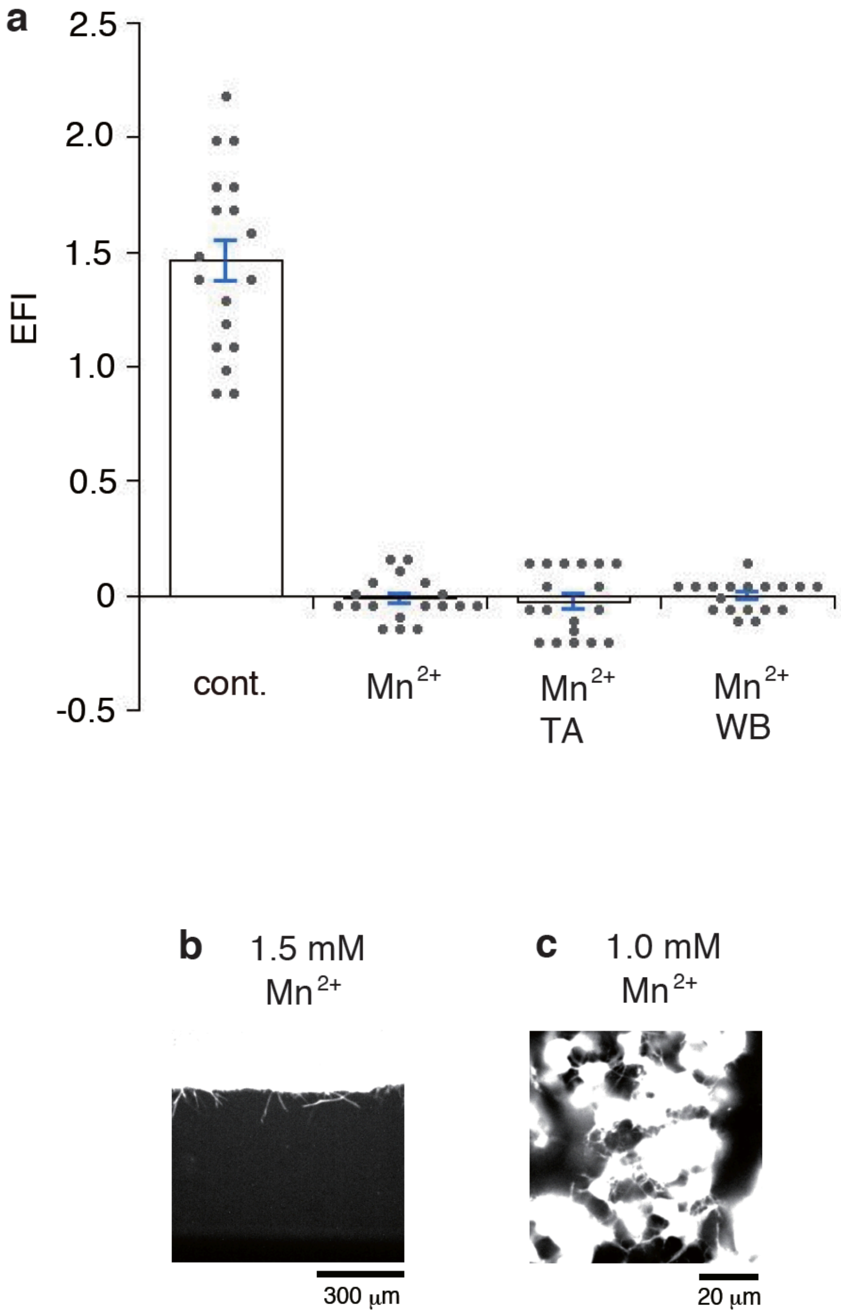

Supplementary Fig. 2: Mn^{2+} abolishes EF effects and high Mn^{2+} suppresses RGC axon outgrowth.

a EFIs (mean ± s.e.m.) of retinal strips cultured with 500 μM Mn^{2+} in the ventrally directed EF without antibodies (Mn^{2+}), with TASC (Mn^{2+} TA) and W1B10 (Mn^{2+} WB). The control without Mn^{2+} (cont.) was replotted from Supplementary Fig. 1e (cont.). The other data were obtained from Fig. 2g. **b** RGC axons cultured with 1.5 mM Mn^{2+} in the ventrally directed EF. **c** RGCs with local arborizations cultured with 1.0 mM Mn^{2+} without EF.

Supplementary Figure 3

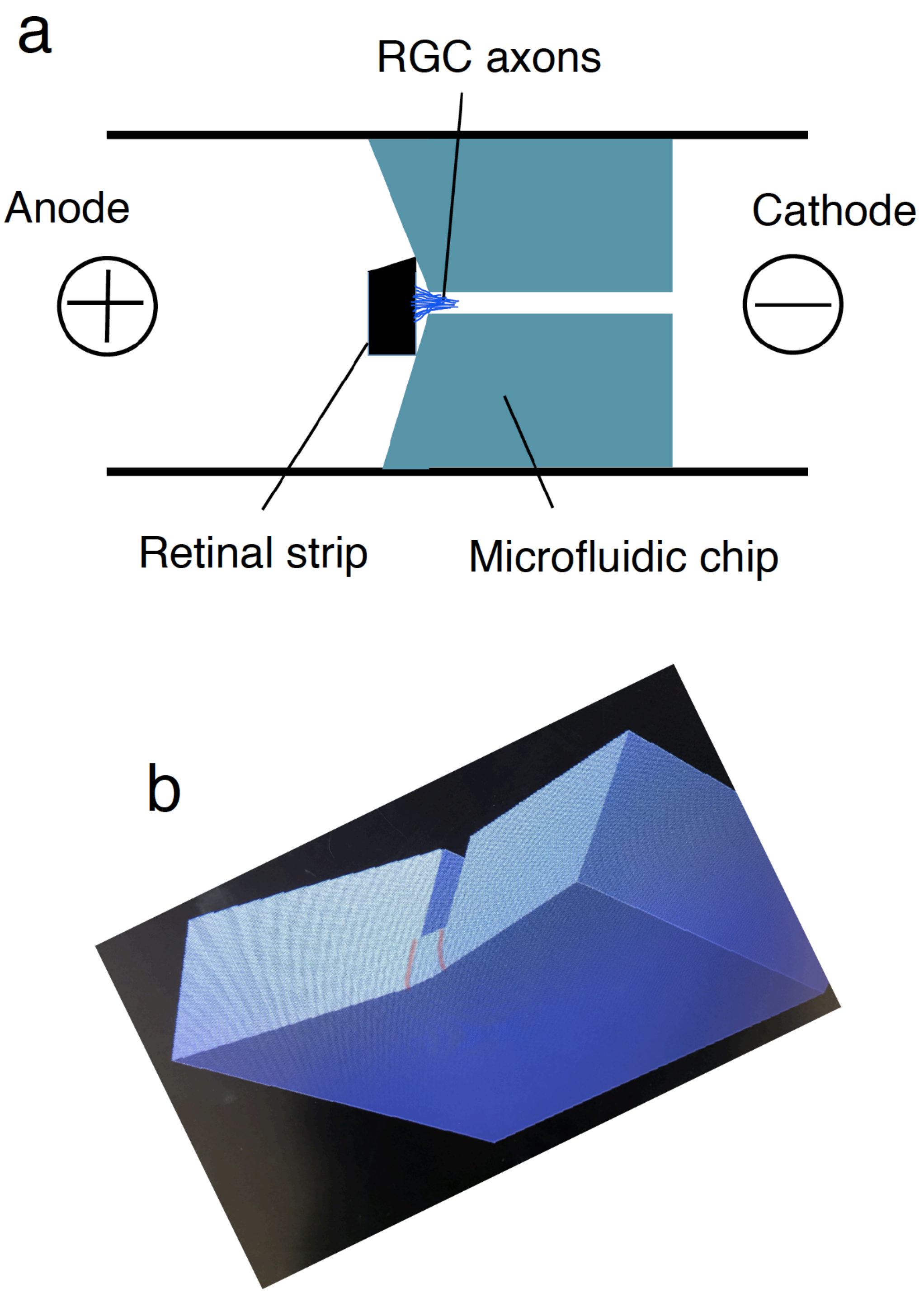

Supplementary Fig. 3: Fan-shaped microfluidic chip.

a A schematic drawing of the whole setup of retinal strip and microfluidic chip. **b** 3-D image of microfluidic chip from the bottom. Dimensions are described in Methods.

Supplementary Figure 4

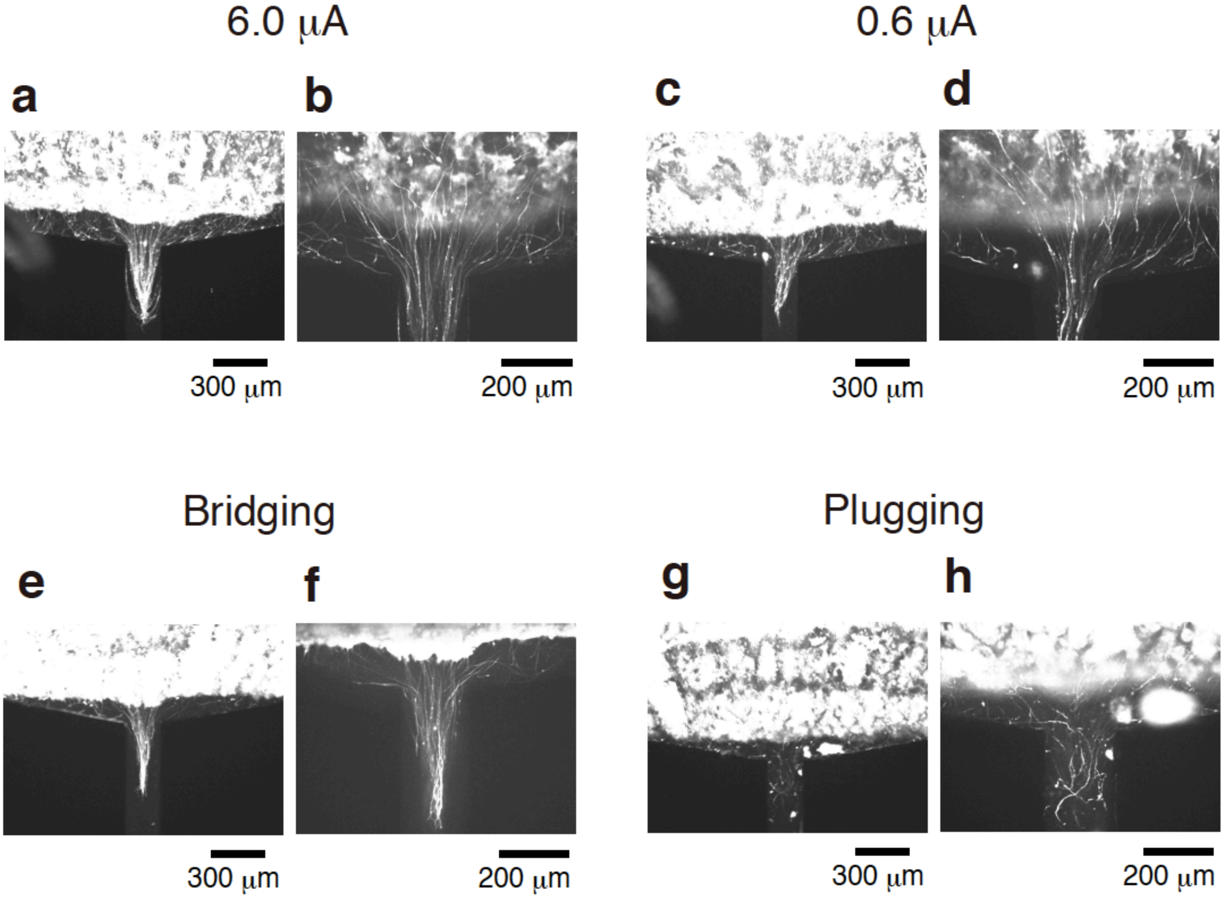

Supplementary Fig. 4: RGC axon convergence in weak focused EFs.

a-b A retinal strip cultured in the focused EF by 6.0 μA. **a** The ventral side of the retinal strip facing the open side of a fan-shaped microfluidic chip with the central channel, through which a current of 6.0 μA flowed to the cathode. The outgrowing axons converged on the channel and entered it. **b** A high-magnification image of the converging axons in **a**. **c-d** A retinal strip cultured in the focused EF by 0.6 μA. **c** The ventral side of the retinal strip. **d** A high-magnification image of **c**. The EF strength by applying 6.0 μA was estimated as 1 mV/mm around the retinal strip and that at the entrance of the channel was 30 mV/mm (see Methods). Those by 0.6 μA were 0.1 mV/mm and 3 mV/mm, respectively. **e-f** A retinal strip cultured without exogenous current. The exit of the channel was connected to the culture medium of excess volume with a U-shaped glass tube bridge filled with the culture medium. **e** The ventral side of the retinal strip. **f** A high-magnification image of **e**. **g-h** A retinal strip cultured without exogenous current. The exit of the channel was blocked with a rubber plug. **g** The ventral side of the retinal strip. **h** A high-magnification image of **g**.

Supplementary Figure 5

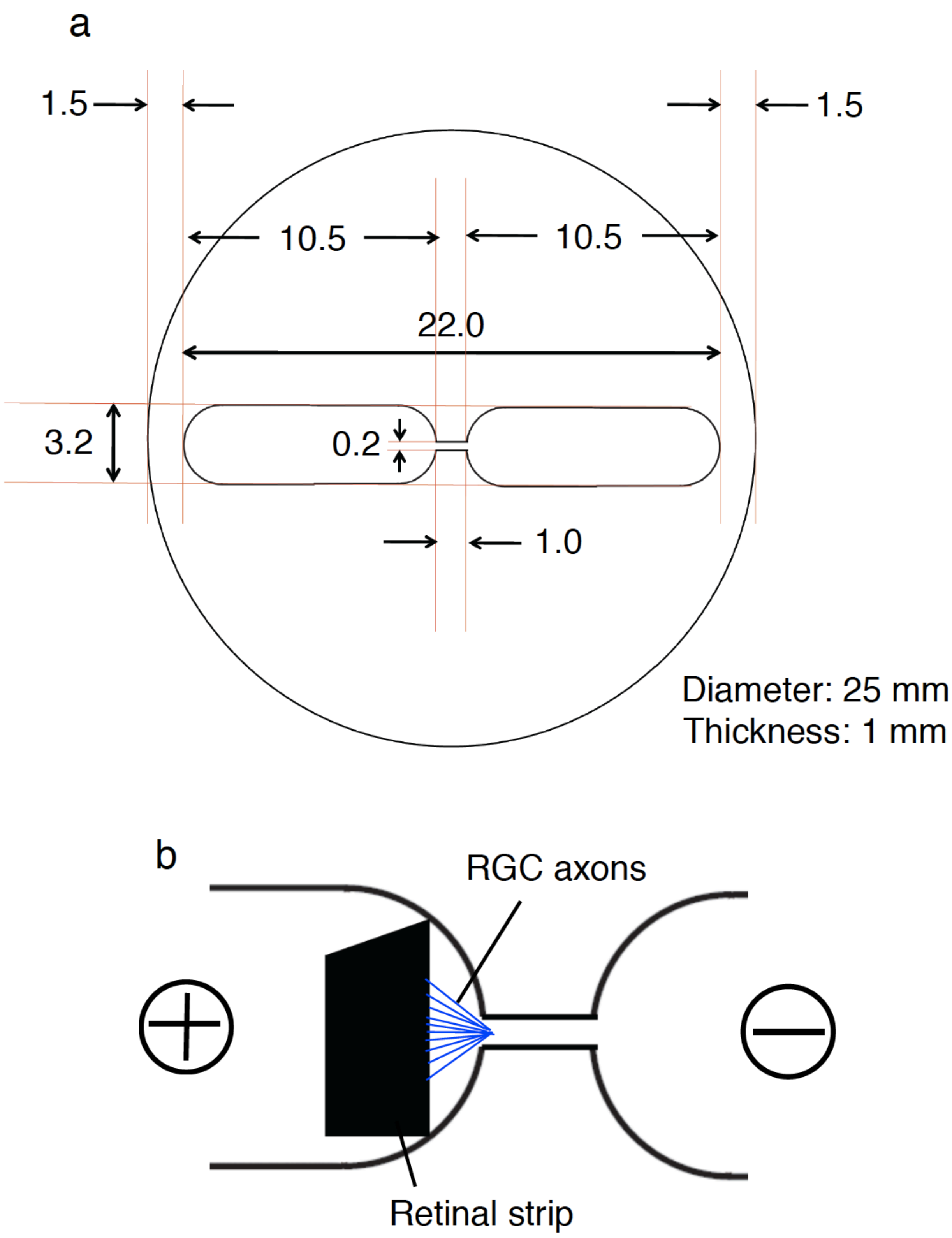

Supplementary Fig. 5: Microchannel chamber.

a Dimensions of the microchannel chamber. **b** A schematic drawing of the whole setup of retinal strip and microchannel. The ventral edge of retinal strip faced the microchannel.

Supplementary Figure 6

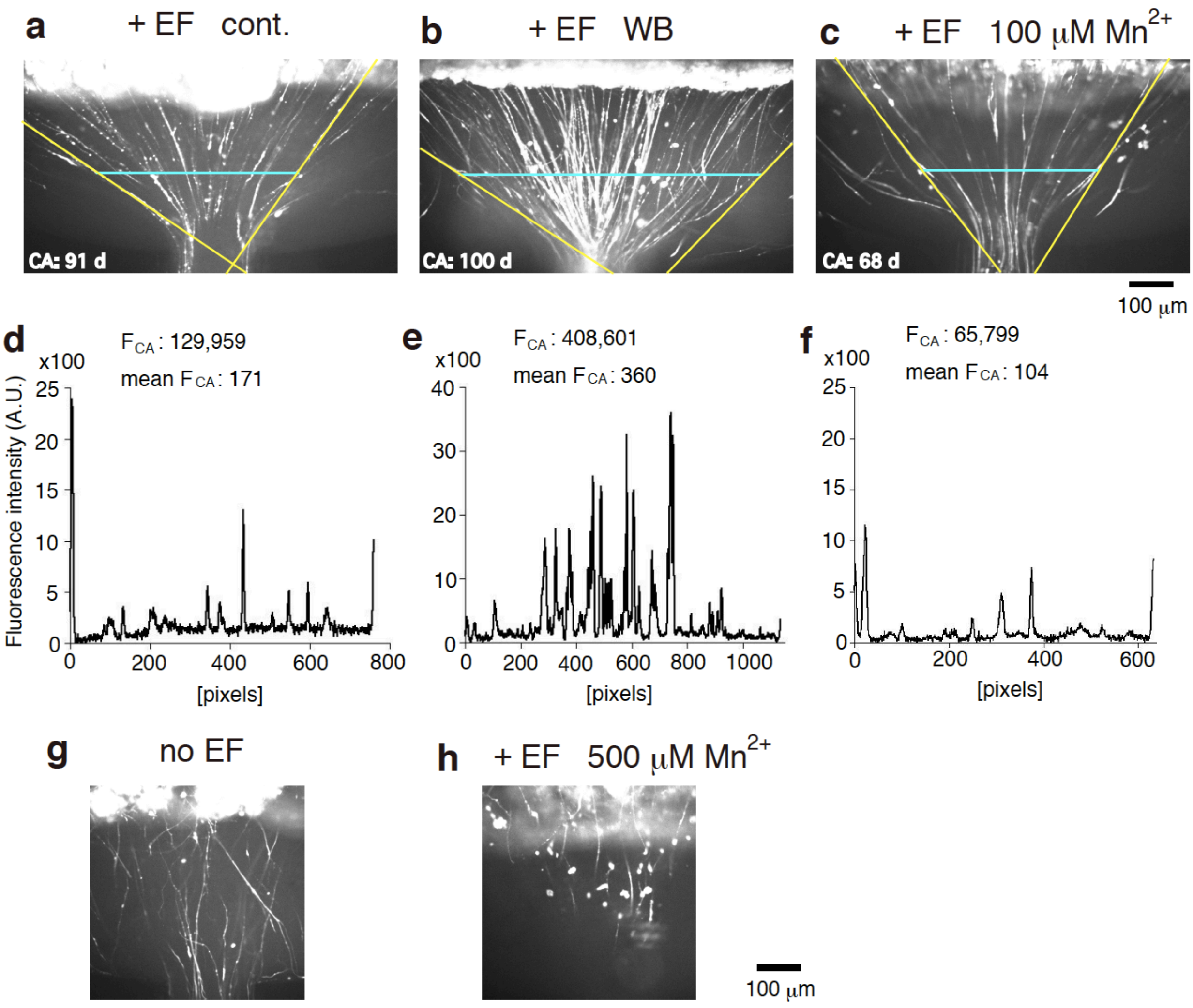

Supplementary Fig. 6 Convergence angle (CA) and fluorescence intensities within CA (F_{CA}).

a-c The fluorescence images in Fig. 3a-c. Yellow lines were drawn on the axons extending straight from the most nasal and temporal regions of the retinal strip. The cyan line indicates the half-distance line between the retina and the microchannel. **d-f** Transverse profiles of fluorescence intensities at the half-distance line in **a-c**. F_{CA} is the total sum of transverse profile. Mean F_{CA} was obtained by dividing F_{CA} with the number of pixels. One pixel size: 650 x 650 nm. **g** RGC axons cultured without EF. **h** RGC axons cultured with EF and 500 μM Mn^{2+}.

Supplementary Figure 7

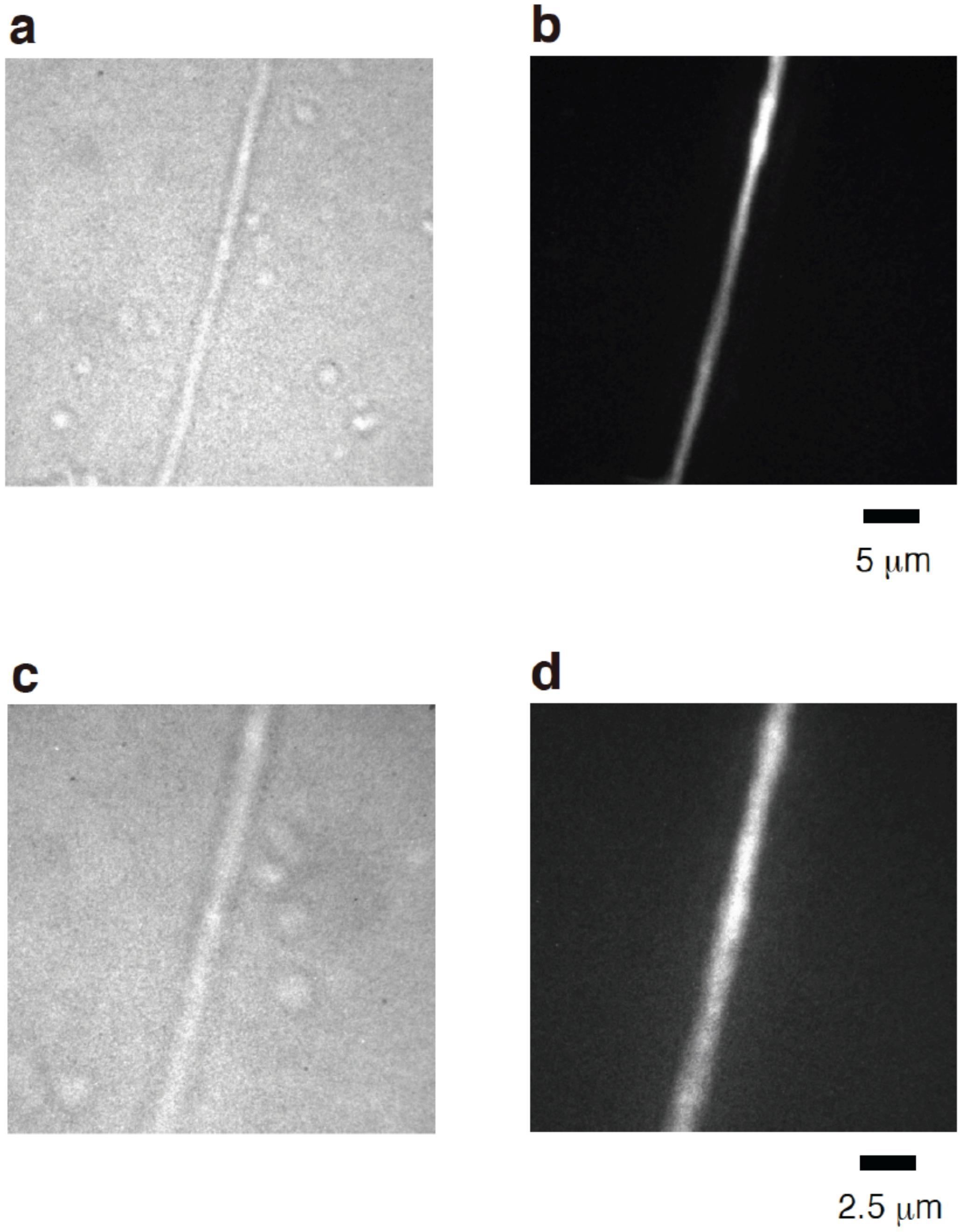

Supplementary Fig. 7: Identification of a single RGC axon with transmitted light.

a A transmission image of an RGC axon in Matrigel®-based thin layer dissociated culture. Aperture stop was most squeezed to increase contrast. **b** A fluorescence image of the axon in **a** stained with calcein-AM. **c-d** Magnified (2x) images of **a** and **b**.

Supplementary Figure 8

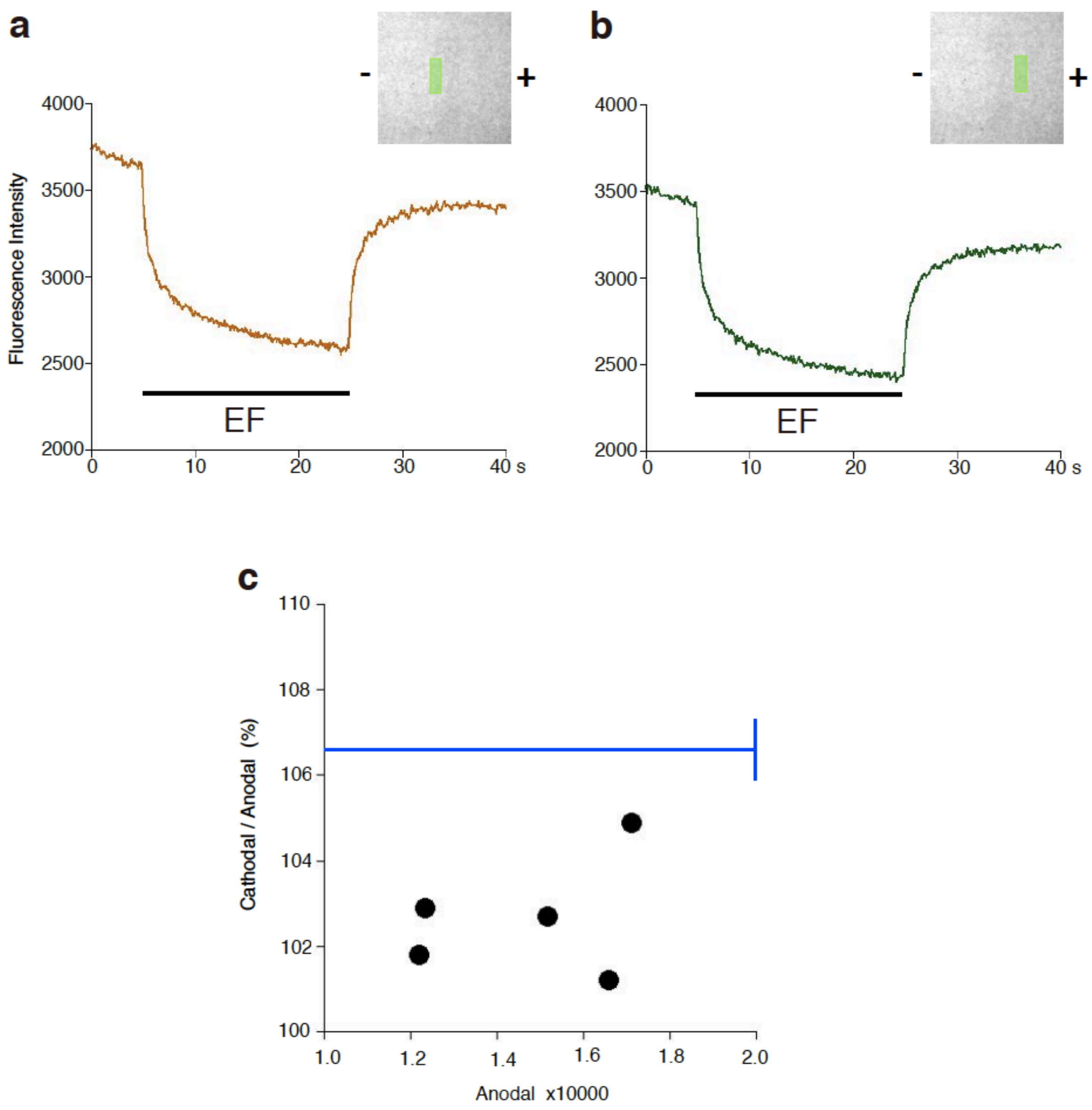

Supplementary Fig. 8: Ca^{2+} fluorescence responses to reverse EF.

a-b Ca^{2+} fluorescence recordings from the cathodal (**a**) and anodal (**b**) sides of an axon. Insets show each recording area. A micropipette containing 1 mM $CaCl_2$ and 10 mM Calbryte^{TM}-520L was positioned at the left (cathodal) side of the axon. The reverse test EF was applied from the right (the anode at the right). The fluorescence intensity is presented with 64-bit depth. **c** Cathodal/anodal ratio of integral values of decreases in fluorescence intensity during EF (fluorescence intensity x time) plotted against the anodal values measured on five RGC axons. The horizontal blue line indicates the mean cathodal/anodal ratio of the fluorescence intensities before EF application (vertical line represents ± s.e.m.).

Supplementary Figure 9

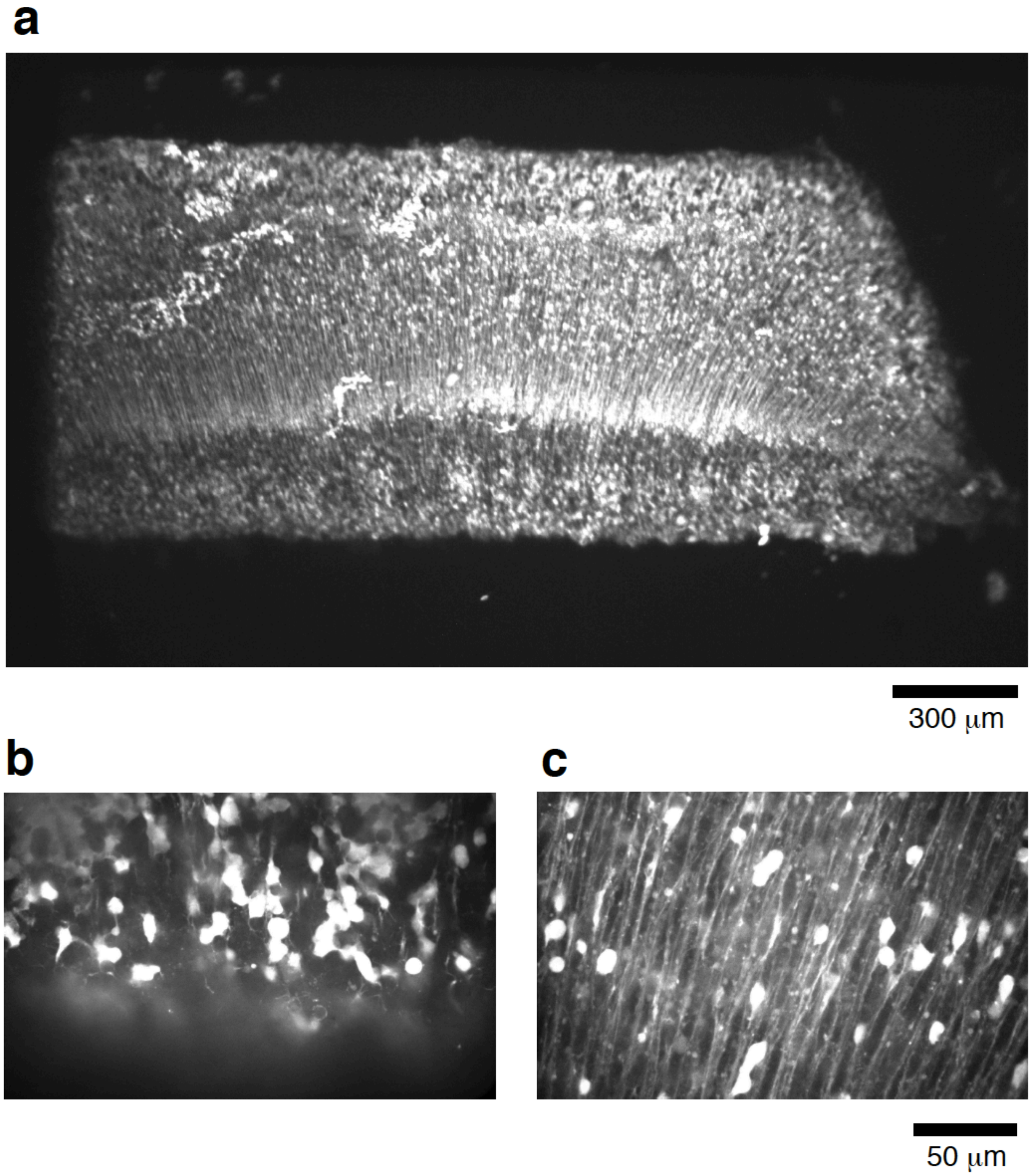

Supplementary Fig. 9: Cytochalasin D prevents RGC axon outgrowth.

a A retinal strip cultured for 24 hours with cytochalasin D (0.5 μg/mL, approximately 1 μM). **b** The ventral edge of the retinal strip in **a**. **c** RGC axons in the optic fiber layer of the retinal strip in **a**.

Supplementary Figure 10

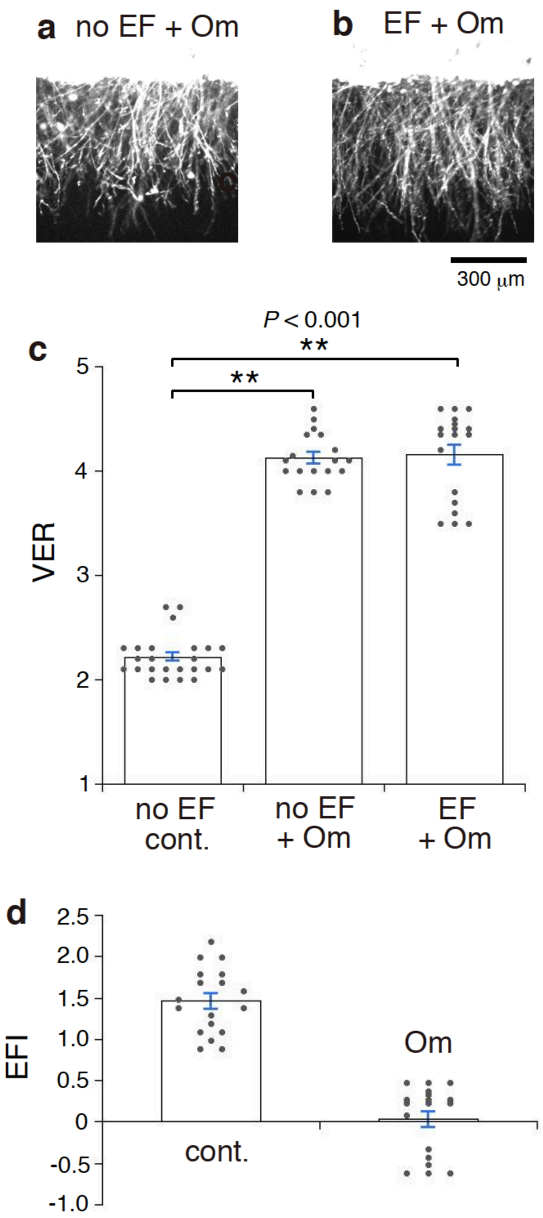

Supplementary Fig. 10: Inhibition of phosphatidylinositol-3 kinase (PI3K) enhances RGC axon extension without EF and abolishes EF effects.

a RGC axons cultured without EF in the presence of an inhibitor of PI3K, omipalisib at 2.5 nM. **b** RGC axons cultured with 2.5 nM omipalisib in the ventrally directed EF. **c** VERs (mean ± s.e.m.) of retinal strips cultured in the presence of 2.5 nM omipalisib without EF (no EF + Om, n = 18 from 3 retinal strips) and with EF (EF + Om, n = 18 from 3 retinal strips). The control without drug (no EF cont.) was replotted from Fig. 1k (no). Horizontal bars and asterisks denote significant differences (two-tailed t-test, ** $P < 0.001$). **d** EFIs (mean ± s.e.m.) of retinal strips cultured without drug (cont.) and with omipalisib in the ventrally directed EF. The control was replotted from Supplementary Fig. 1e (cont.).

Supplementary Figure 11

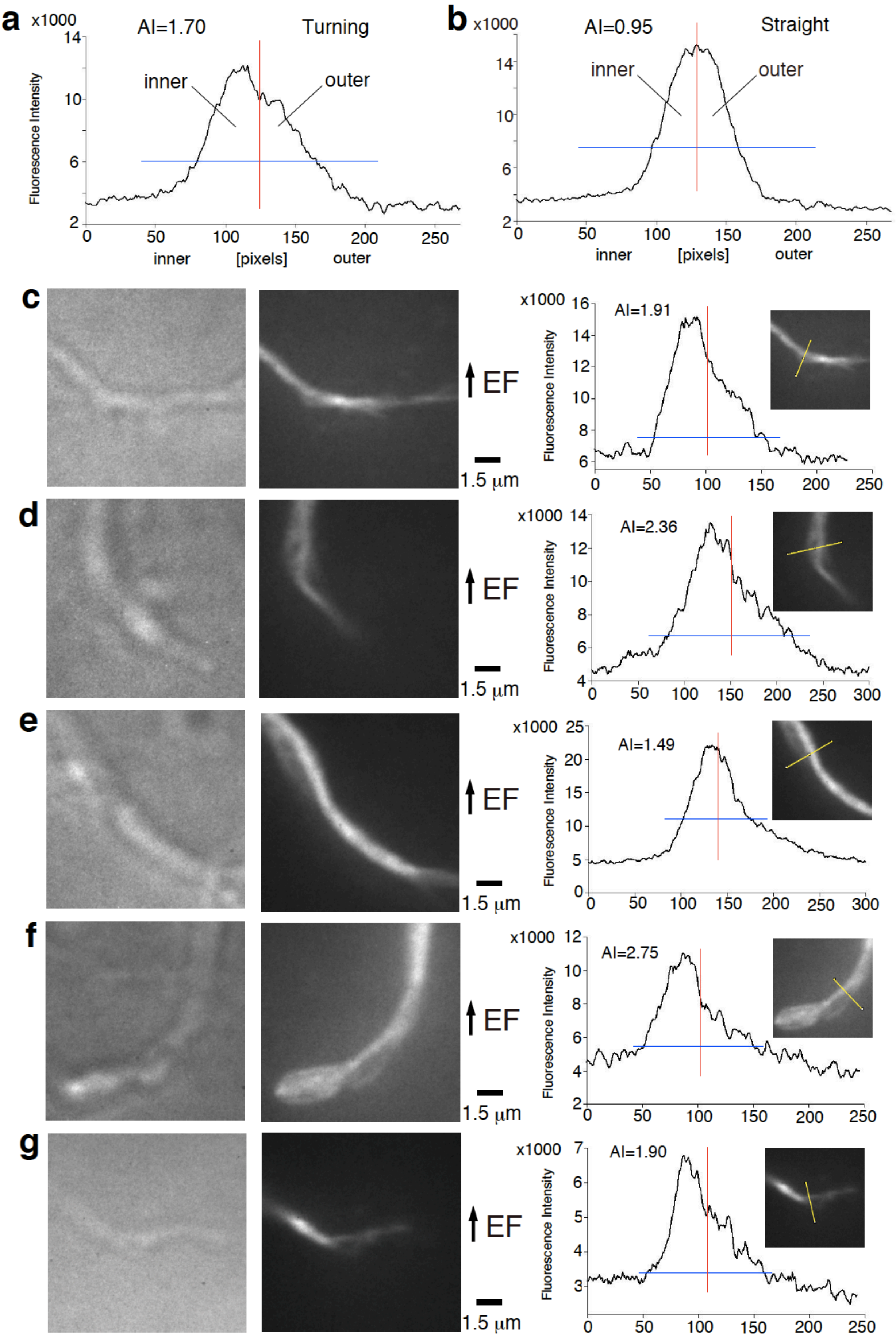

Supplementary Fig. 11: Asymmetry index (AI) of microtubule distribution.

a-b Transverse profiles of the fluorescence image in Fig. 5l at the turning point (**a**) and the straight part (**b**). The horizontal blue line indicates the half level of the peak intensity. The vertical red line indicates the middle point of the half-width. The asymmetry index (AI) was the ratio of the total fluorescence intensities at the inner (cathodal) side against that at the outer (anodal) side above the half level. **c-g** Transmission and fluorescence images and transverse profile at turning point (yellow line in inset) of five RGC axons turning dorsally in the dorsally directed EF. **f** Background intensity (3449) was subtracted in the transverse profile. **g** An RGC axon stained with TubulinTracker^{TM} Green after 30 min of nocodazole treatment (5 μg/mL). One pixel size: 14.4 x 14.4 nm.

Supplementary Figure 12

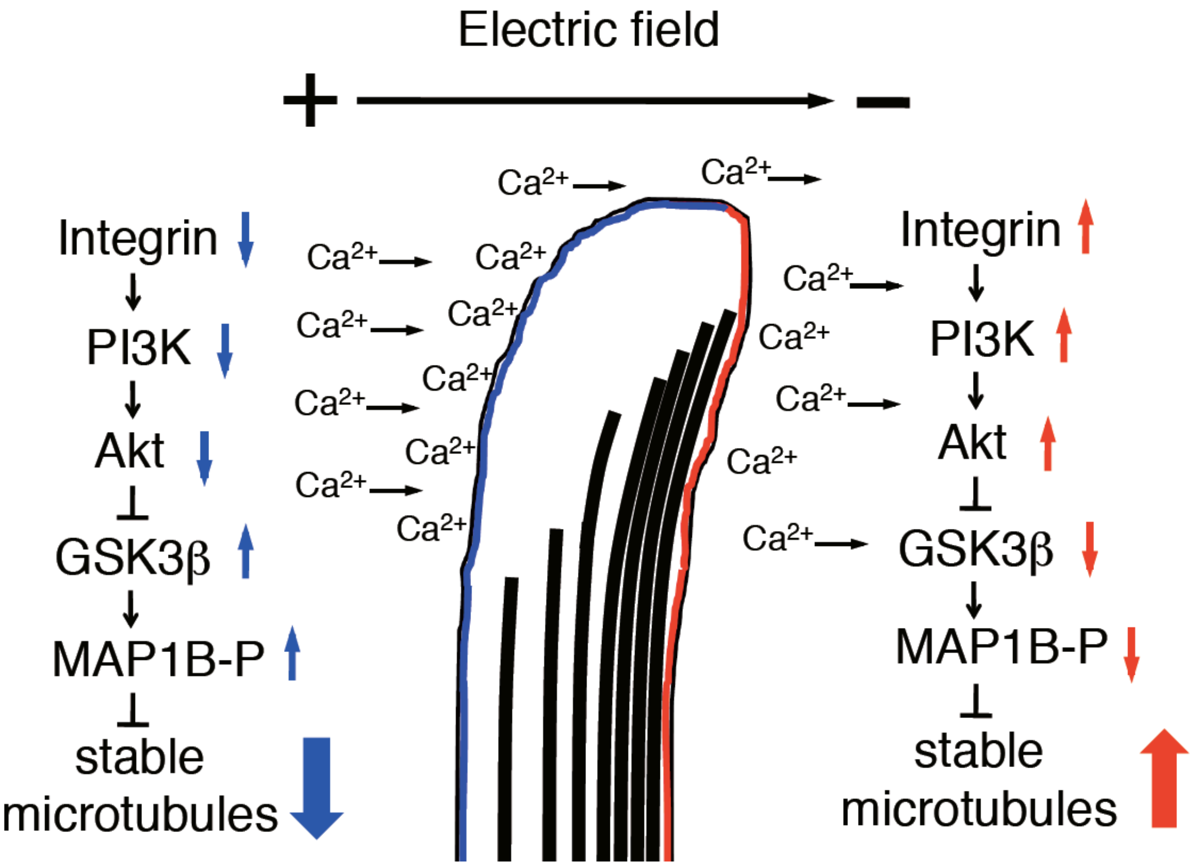

Supplementary Fig. 12: A proposed model for electric axon guidance.

The anodal surface of an axon encounters EF-moved Ca^{2+} more frequently than the cathodal surface. As Ca^{2+} binding to ADMIDAS in the ligand-binding domain of β subunit inhibits integrin-ligand binding, integrin is more inhibited on the anodal side than on the cathodal side. Since the activation of integrin stabilizes microtubules through PI3K activation, Akt activation, and GSK3β inhibition, the less inhibited integrin on the cathodal side stabilizes more microtubules. As an axon turns in the direction of stabilized microtubules, the axon turns toward the cathode.

Supplementary Figure 13

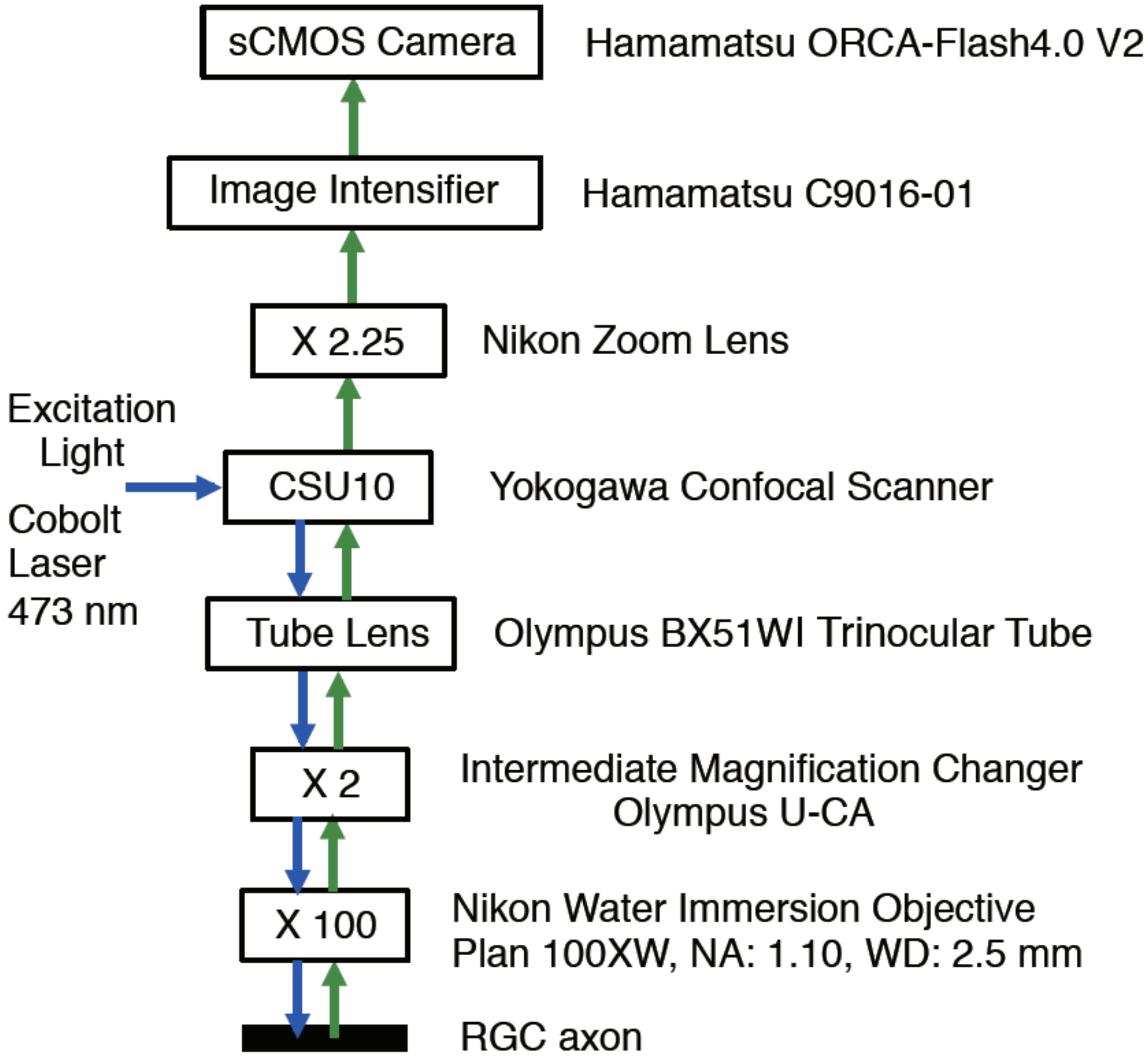

Supplementary Fig. 13: High-magnification confocal microscope system.
Two magnifying lenses are inserted in the light path to obtain a magnification of 14.4 nm x 14.4 nm/pixel. An image intensifier was connected to the sCMOS camera. A high-power laser source (up to 150 mW) was used.
