# Supplementary material for: Integrin-mediated electric axon guidance underlying optic nerve formation in the embryonic chick retina
Source: Commun Biol. 2023 Jun 30;6:680. doi: 10.1038/s42003-023-05056-x (PMC10313674; doi:10.1038/s42003-023-05056-x)
Supplement: Supplementary file 3 — Description of Additional Supplementary Files [file 42003_2023_5056_MOESM3_ESM.docx]

**Description of Additional Supplementary Files**

**File name:** Supplementary Data 1
**Description:** Source data for Fig. 1d, 1k-l, 2g, 3d-f, 4d-g, 5i, 5m, 5j.
